# Association between type 2 diabetes mellitus and depression among Korean midlife women: a cross-sectional analysis study

**DOI:** 10.1186/s12912-023-01385-8

**Published:** 2023-07-10

**Authors:** You Lee Yang, Eun-Ok Im, Yunmi Kim

**Affiliations:** 1 College of Nursing, Eulji University, 553, Sanseong-daero, Sujeong-gu, Seongnam-si, Gyeonggi-do 13135 Republic of Korea; 2 School of Nursing, Emory University, 1520 Clifton Road, Atlanta, GA USA

**Keywords:** Depression, Diabetes mellitus, Type 2, Midlife, Women

## Abstract

**Background:**

The prevalence of depression is higher among midlife women, and they have less control over their diabetes during the menopausal transition. However, there is limited evidence on the association between type 2 diabetes mellitus and depression among Korean women in their midlife. This study aimed to examine the association between type 2 diabetes mellitus and depression and explore the levels of awareness and treatment of depression among Korean midlife women with T2DM.

**Methods:**

This is a cross-sectional analysis study conducted using data from the Korea National Health and Nutrition Examination Surveys of 2014, 2016, and 2018. Korean women aged 40–64 years who randomly participated in the surveys were included, and 4,063 midlife women were selected as study participants. The diabetes progression status of the participants was classified into diabetes, pre-diabetes, and non-diabetes. Furthermore, the Patient Health Questionnaire-9 was used for screening depression. Participants’ awareness rate, treatment rate among incident cases of depression, and treatment rate among awareness cases of depression were also analyzed. For data analysis, the Rao–Scott χ2 test, multiple logistic regression, and linear regression were conducted using SAS 9.4 software program.

**Results:**

The prevalence of depression significantly differed between diabetes, pre-diabetes, and non-diabetes groups. However, depression awareness, treatment/incident, and treatment/awareness rates did not differ statistically between the diabetes progression status groups. Compared to the non-diabetes group, diabetes group had a higher odds ratio of depression after adjusting for general and health-related factors. Thus, the diabetes group had significantly higher PHQ-9 scores than the non-diabetes group after adjusting for covariates.

**Conclusions:**

Women in their midlife who have type 2 diabetes mellitus tend to have higher levels of depressive symptoms and are at risk of depression. However, we found no significant differences between diabetes and non-diabetes regarding the awareness and treatment rates of depression in South Korea. We recommend that future studies focus on developing clinical practice guidelines aimed at additional screening and intervention for depression in midlife women with type 2 diabetes mellitus to ensure prompt treatment and improved outcomes.

## Background

Type 2 diabetes mellitus (T2DM) and depression are major health concerns worldwide [1–3]. In 2021, the Korea Diabetes Association reported that overall prevalence of T2DM was approximately 13.9%, which is higher than the globally reported prevalence of 10% [4, 5]. Specifically, among Korean women, 6.8% aged 40–49 years, 10.1% aged 50–59 years, and 20.9% aged 60–69 years have T2DM, and women tend to have a lower glucose control rate than men [4]. Additionally, the prevalence of depression in South Korea was reported to be significantly higher in women (6.8%) than in men (3.9%), higher than the global rate of 4.4% (5.1% and 3.6% in women and men, respectively) [6, 7].

Prior studies indicate that depression has a bi-directional relationship with T2DM. Depression increases the risk of developing diabetes because antidepressants negatively affect insulin sensitivity and glycemic control [8]. Moreover, people with diabetes are twice as likely to be depressed as people without diabetes because of the psychological burden of being ill, or because of an unfavorable lifestyle such as lack of physical activity, unhealthy diet, or stressful lifestyle [9]. In one study, depressive symptoms related to diabetes complications such as renal failure, neuropathy, and foot ulcers were closely associated with higher readmission and mortality rates [2]. Furthermore, Chang and Im [10] proposed that depression is a psychosocial factor affects health-related quality of life among older South Korean adults with T2DM. Both T2DM and depression decrease the quality of life and the negative impact of T2DM and depression combined may be more significant [11].

Depression usually remains underdiagnosed and untreated; thus diabetes screening and the awareness of depression in diabetes care are important. A recent systematic review reported that the Patient Health Questionnaire-9 (PHQ-9) is the most used and validated screening tool for depression and diabetes [12]. The PHQ-9 records the frequency of depressive symptoms using 9 items over the past 2 weeks. Scores of 10 or higher (out of 27) are commonly regarded as an indicator of depression in clinical studies, suggesting that treatment is required [13].

Midlife women often experience depression, which is a mental health condition that contributes to T2DM [14, 15]. Given that menopause is associated with both T2DM and depression, menopausal midlife women are particularly vulnerable to T2DM and depression [16]. Hormonal changes in menopause transition can lead to glucose metabolic imbalance and the development of depressive symptoms in women [16, 17]. The Study of Women’s health Across the Nation (SWAN) in the USA indicates that women are at greater risk of developing depressive symptoms during the menopausal transition, especially in the postmenopausal period [16, 18].

Social roles in Korean culture often require midlife women to prioritize their family’s needs over their own health and well-being, potentially making it more difficult to manage depression and T2DM [9]. Previous qualitative studies suggest that Korean midlife women with T2DM face difficulties caring for their parents-in-law, experience suicidal ideation, sacrifice themselves for their family or colleagues, and see diabetes care as their own responsibility [19]. These experiences may negatively impact their quality of life and their ability to manage their diabetes, leading to worsening depressive symptoms.

To our knowledge, despite the high prevalence of T2DM and depression in Korean midlife women, no studies have explored the association between T2DM and depression or the awareness and treatment rate of depression among Korean midlife women with T2DM. Identifying this association could provide significant clinical insight into the care of these patients and help guide the development of better approaches for managing diabetes and depression. It could also help identify risk factors for depression in this group of women, allowing for early intervention and improved outcomes.

Therefore, the aims of this study are as follows: (1) to identify differences in the T2DM progression status in Korean midlife women with or without depression, (2) to investigate the differences in awareness and treatment rate of depression based on the T2DM progression status in women who were screened for depression (PHQ-9 ≥ 10), and (3) to explore the association between T2DM progression status and depression in Korean midlife women.

## Methods

We conducted a cross-sectional analysis study using the data from the Korea National Health and Nutrition Examination Survey (KNHANES) in 2014, 2016, and 2018. Before conducting this study, we obtained approval from the institutional review board (IRB) of the author’s affiliated institution (EUIRBN2020-043).

### Data source and participants

The KNHANES is a nationwide cross-sectional survey conducted annually by the Korea Ministry of Health and Korea Centers for Disease Control and Prevention to monitor the general health and nutritional status of Koreans. All survey participants are Korean citizens recruited using a randomized and multi-stage cluster sampling method considering their residential area, sex, and age. Thus, this survey is representative of the Korean population and provides a comprehensive view of their health status including health behavior, awareness, physical assessments, and clinical test results (e.g., blood test for HbA1c). Furthermore, KNHANES adheres to international standards, with a 75% response rate for statistical analysis to ensure the accuracy and quality of data; therefore, it best reflects the general and health-related characteristics of the Korean population [20].

**Fig. 1:**
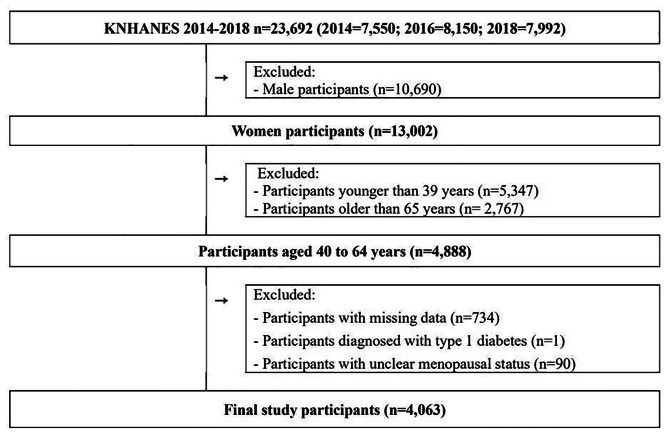
Flowchart of study participant selection. Of the 23,692 participants, male participants (n = 10,690), participants younger than 39 years (n = 5,347), participants older than 65 years (n = 2,767), participants with missing data (n = 734), participants diagnosed with type 1 diabetes (n = 1), and participants with unclear menopausal status (n = 90) were excluded

In this study, we used data from 2014, 2016, and 2018 KNHANES surveys, which adopted the globally recognized and reliable PHQ-9 screening tool for depression [21]. Figure 1 displays the details of the study participant selection process. Of the 23,692 participants of KNHANES 2014, 2016, and 2018, our primary selection included 4,888 women aged 40–64 years. We excluded participants with missing data (n = 734), participants diagnosed with type 1 diabetes (n = 1), and participants having unclear menopausal status (n = 90). A total of 4,063 participants were included in the final data analysis. According to G*Power 3.1.9.7 program [22], the statistical power (1-β) of multiple regression analysis was calculated as > 0.99 with effect size (f^2^) 0.10, significance level (α) 0.05, and the number of predictors 15.

### Variables

T2DM and depression were examined as major disease variables. Based on the World Health Organization (WHO) and American Diabetes Association (ADA) diagnostic criteria [23, 24], we categorized patients into diabetes, pre-diabetes, and non-diabetes. Those who had Fasting Plasma Glucose (FPG) ≥ 126 mg/dL, HbA1C ≥ 6.5%, were diagnosed as patients with diabetes by a doctor; those who were taking diabetes medication or insulin injections were also considered patients with diabetes. Those who had FPG ≥ 100 mg/dL to ≤ 125 mg/dL or HbA1c ≥ 5.7% to ≤ 6.4% were considered pre-diabetes, and those who had FPG < 100 or HbA1C ≤ 5.7% were non-diabetes. Moreover, we classified depression as severe for those with scores of 10 or higher on the PHQ-9 Korean version [13, 25].

Participants’ general and health-related characteristics were set as variables in this study. From the literature review, we considered the following variables as potential covariates [16, 17]. For general characteristics, age (40–49, 50–59, or 60–64 years), marital status (married/partnered or nonmarried/separated), family income (above average or below average), residential region (urban or rural), education attained (college or higher, high school, or middle school), employment status (unemployed or employed) were included as covariates. For health-related characteristics, alcohol consumption frequency (never or < 1 time per month, 1–4 times per month, or ≥ 1 time per week), smoking status (never, ex-smoker, smoker), Asia-Pacific body mass index (kg/m^2^) (underweight [< 18.5], normal [18.5–22.9], pre-obese [23.0–24.9], or obese [≥ 25.0]), menopausal status (post-menopausal or pre-menopausal), childbirth experience (no or yes), practicing aerobic exercise (no or yes), and restriction in activity (no or yes) were included as covariates. In determining menopausal status, women who had stopped menstruating or had undergone a hysterectomy were classified as post-menopausal. Women with depression were classified into awareness and treatment cases based on whether they had been diagnosed with depression by a doctor.

### Statistical analyses

To ensure the representativeness of the Korean population, all analyses were based on the sampling weight, cluster, and strata, which were generated based on the sample design. The Rao–Scott chi-squared test (χh) was performed to identify the differences in general and health-related characteristics between study participants with or without depression. It also examined the differences in awareness, treatment, and treatment/awareness rates of depression between diabetes status among those with depression. Multiple logistic regression was used to calculate the adjusted odds ratios (ORs) for depression according to the presence of diabetes while controlling for confounding variables. Linear regression models were used to identify the associations between diabetes and PHQ-9 scores while controlling for confounding variables. The SAS 9.4 (SAS Institute, Cary, NC) software program was used for data analysis. We assigned significance level *p* < .05 and calculated weighted frequencies and percentages to generalize the KNHANES data.

## Results

### Differences in general and health-related characteristics of depression

Among the study participants (n = 4,063), 261(6.5%) had depression. T2DM was diagnosed in 416 (10.2%) participants. Table 1 presents the differences between those with and without depression regarding general and health-related characteristics. Marital status (χh = 59.72), family income (χh = 40.65), education attained (χh = 31.53), economic activity (χh = 28.16), drinking frequency (χh = 6.03), smoking status (χh = 52.70), menopausal status (χh = 8.12), and restriction in activity (χh = 245.28) showed significant differences between those with and without depression (*p* < .05). Additionally, there was a significant difference in the prevalence of depression between groups of diabetes (12.5%), pre-diabetes (6.2%), and non-diabetes (5.6%) (χh = 19.17, *p* < .001).

**Table 1:** Background characteristics and health status by depression (n = 4,063)

Characteristics (Mean ± SD)	Categories	Total	Depression		χ*h*	*p*
			No (n = 3,802, 93.5%)	Yes (n = 261, 6.5%)		
		n(%)	n(%)	n(%)		
Age (52.03 ± 7.09)	40–49	1,612(39.7)	1,527(94.7)	85(5.3)	4.24	0.120
	50–59	1,682(41.4)	1,568(93.2)	114(6.8)		
	60–64	769(18.9)	707(91.9)	62(8.1)		
Marital status	Married or partnered	3,404(83.8)	3,236(95.1)	168(4.9)	59.72	< 0.001
	Nonmarried or separated	659(16.2)	566(85.9)	93(14.1)		
Family income	Above average	2,632(64.8)	2,521(95.8)	111(4.2)	40.65	< 0.001
	Below average	1,431(35.2)	1,281(89.5)	150(10.5)		
Residential region	Urban	3,387(83.4)	3,177(93.8)	210(6.2)	1.13	0.287
	Rural	676(16.6)	625(92.5)	51(7.5)		
Education attained	College or higher	1,220(30.0)	1,177(96.5)	43(3.5)	31.53	< 0.001
	High school	1,667(41.0)	1,572(94.3)	95(5.7)		
	Middle school	1,176(28.9)	1,053(89.5)	123(10.5)		
Employment	Unemployed	1,577(38.8)	1,429(90.6)	148(9.4)	28.16	< 0.001
	Employed	2,486(61.2)	2,373(95.5)	113(4.5)		
Drinking frequency	Never or < 1*per month 1–4*per month ≥ 1*per week	2,330(57.3)	2,170(93.1)	160(6.9)	6.03	0.049
		1,246(30.7)	1,187(95.3)	59(4.7)		
		487(12.0)	445(91.4)	42(8.6)		
Smoking status	Never smoked Ex-smoker Smoker	3,690(90.8)	3,492(94.6)	198(5.4)	52.70	< 0.001
		168(4.1)	140(83.3)	28(16.7)		
		205(5.0)	170(82.9)	35(17.1)		
Body mass index, kg/m^2^	Underweight(< 18.5)	129(3.2)	116(89.9)	13(10.1)	3.96	0.266
	Normal(18.5–22.9)	2,420(59.6)	2,286(94.5)	134(5.5)		
	Pre-obese(23.0-24.9)	1,118(27.5)	1,035(92.6)	83(7.4)		
	Obese(≥ 25.0)	396(9.7)	365(92.2)	31(7.8)		
Menopausal status*	Pre-menopausal	1,749(43.0)	1,661(95.0)	88(5.0)	8.12	0.004
	Post-menopausal	2,314(57.0)	2,141(92.5)	173(7.5)		
Childbirth experience	No	199(4.9)	177(88.9)	22(11.1)	1.45	0.229
	Yes	3,864(95.1)	3,625(93.8)	239(6.2)		
Practicing Aerobic exercise	No	2,227(54.8)	2,074(93.1)	153(6.9)	0.16	0.688
	Yes	1,836(45.2)	1,728(94.1)	108(5.9)		
Restriction on Activity	No	3,801(93.6)	3,629(95.5)	172(4.5)	245.28	< 0.001
	Yes	262(6.4)	173(66.0)	89(34.0)		
Diabetes status	Normal	2,070(50.9)	1,957(94.5)	113(5.5)	19.17	< 0.001
	Pre-diabetes	1,577(38.8)	1,473(93.4)	104(6.6)		
	Diabetes	416(10.2)	372(89.4)	44(10.6)		

Note, n: unweighted sample size; p-value was obtained using a Rao–Scott χ^2^ test based on weighted percentage. *Those who underwent a hysterectomy were categorized as post-menopausal

### Awareness, treatment/incident, and treatment/awareness cases in depression by diabetes progression status

Among the 261 participants who had depression, 112 (42.9%) were aware that they had depression, and 87 (33.3%) were receiving treatment for depression. Among the 112 participants who were aware of their depression, 87 (77.7%) were receiving treatment. However, there were no significant differences in the awareness and treatment of depression by diabetes progression status (*p* > .05) (Table 2).

**Table 2:** The differences in the awareness, treatment/incident, and treatment/awareness cases in depression by diabetes progression status (n = 261, 5.3%)

Categories		Total	Non-diabetes (n = 94, 2.3%)	Pre-diabetes (n = 88, 1.9%)	Diabetes (n = 40, 1.1%)	χ*h*	*p*
		n (%)	n (%)	n (%)	n (%)		
Awareness	Yes	112 (42.9)	46 (40.7)	43 (41.3)	23 (52.3)	0.23	0.892
	No	149 (57.1)	67 (59.3)	61 (58.7)	21 (47.7)		
Treatment /incident cases	Yes	87 (33.3)	38 (33.6)	31 (29.8)	18 (40.9)	0.22	0.898
	No	174 (66.7)	75 (66.4)	73 (70.2)	26 (59.1)		
Treatment /awareness cases*	Yes	87 (77.7)	38 (82.6)	31 (72.1)	18 (78.3)	0.67	0.715
	No	25 (22.3)	8 (17.4)	12 (27.9)	5 (21.7)		

Note. * Only those who were aware of their depression were included

### Association between depression and T2DM in midlife women

Table 3 shows the association between depression and T2DM in midlife women. According to logistic regression analysis, midlife women with diabetes were significantly more likely to develop depression (OR = 1.69, 95% CI = 1.08–2.66) than those without diabetes, after controlling for general and health-related variables. According to linear regression analysis, diabetes (B = 0.63, *p* = .008) was significant indicator of PHQ-9 scores, and this regression model explained 17.3% of the dependent variable (adjusted R^2^ = 0.173, *p* < .001).

**Table 3:** Factors associated with depression grade and score (n = 4,063)

Characteristics	Categories	Depression		PHQ-9 score	
		OR (95% CI)	*Ρ*	B	*Ρ*
Age, years		0.98 (0.93–1.02)	0.261	-0.05	< 0.001
Marital status	Married or partnered Unmarried or separated	1.00 2.27 (1.59–3.22)	< 0.001	0.00 1.13	< 0.001
Family income	Above average Below average	1.00 1.25 (0.91–1.72)	0.174	0.00 0.63	< 0.001
Residential region	Urban Rural	1.00 1.08 (0.78–1.51)	0.645	0.00 0.19	0.255
Education	College or higher High school Middle school	1.00 1.51 (0.97–2.36) 2.01 (1.17–3.43)		0.00	
			0.070 0.011	0.21 0.61	0.106 0.003
Employment	Unemployed Employed	1.00 0.55 (0.40–0.76)	< 0.001	0.00 -0.57	< 0.001
Drinking	Never or < 1*per month 1–4*per month ≥ 1*per week	1.00 0.73 (0.51–1.06) 1.01 (0.63–1.62)		0.00	
			0.098 0.982	-0.03 0.45	0.804 0.026
Smoking	Never smoked Ex-smoker Smoker	1.00 3.47 (1.89–6.37) 2.14 (1.30–3.51)		0.00 1.60 1.79	
			< 0.001		< 0.001
			0.003		< 0.001
Menopausal status	Pre-menopausal Post-menopausal	1.00 1.22 (0.68–2.17)	0.502	0.00 0.46	0.027
Body mass index, kg/m^2^	Underweight (< 18.5) Normal (18.5–22.9) Pre-obese (23.0–24.9) Obese (≥ 25.0)	1.00 0.77 (0.33–1.79) 0.75 (0.32–1.77) 0.99 (0.38–2.63)	0.539 0.512 0.989	0.00 -0.62 -0.79 -0.85	
					0.073
					0.028 0.034
Childbirth experience	No Yes	1.00 1.52 (0.79–2.94)	0.209	0.00 -0.05	0.878
Practicing aerobic exercise	No Yes	1.00 1.18 (0.87–1.61)	0.283	0.00 -0.06	0.607
Restriction on activity	No Yes	1.00 7.91(5.36–1.66)	< 0.001	0.00 3.99	< 0.001
Diabetes progression status	Non-diabetes Pre-diabetes Diabetes	1.00 0.91 (0.65–1.27)	0.573	0.00 -0.05	0.701
		1.69 (1.08–2.66)	0.022	0.63	0.008

Note. PHQ-9: Patient Health Questionnaire-9

## Discussion

In this study, we found a significant association between T2DM and depression among midlife women in South Korea. The diabetes and non-diabetes groups had no significant difference in depression awareness or treatment cases. Regression analysis showed that T2DM affected depression prevalence and severity after controlling for general and health-related variables. These findings provide evidence on crucial aspects of managing depression in diabetes care for midlife women with T2DM in South Korea.

This large national data sample study showed that the prevalence of depression was 12.5% in Korean midlife women with T2DM, which was significantly higher than the rate of depression found in the pre-diabetes or non-diabetes groups. These results are consistent with previous evidence that women with T2DM are more prone to developing depression, which can contribute to difficulty in managing diabetes [16, 26, 27].

Our study findings revealed that the awareness and treatment rate of depression did not differ significantly between Korean midlife women with and without T2DM. This indicates that participants with T2DM have a higher risk of depression but do not pay attention to their psychological symptoms. According to the study by Im, Yi, and Chee [16], Asian-American women with diabetes had significantly worse depressive symptoms, which put them at a higher risk for depression than White, Black, and Hispanic women. Furthermore, Korean women reportedly experience significant social role strain, which negatively affects the self-management of diabetes [28]. Asian women also tend to have passive attitudes in expressing their somatic symptoms and seeking help from health professionals regarding their psychological distress owing to cultural values [29, 30]. Lifetime depression and anxiety in midlife women are reported to be the strongest predictors of glucose control [31]. These culturally unique characteristics of Korean midlife women with T2DM are crucial factors that may lower the rate of compliance and control of T2DM and accelerate the severity of depressive symptoms. Unlike other countries’ clinical practice guidelines [32, 33], South Korea does not mention depression and/or mental health in its T2DM care guidelines [34, 35]. The results of our study suggest that screening for depressive symptoms with a standardized tool should be part of routine follow-up of Korean midlife women with T2DM. Moreover, providing patients with educational and coaching strategies to control mental health may enhance their treatment and control of the disease.

In other countries, menopausal status has been reported to influence glucose control and depression among midlife women [16, 36]. In our study, menopausal status significantly influenced PHQ-9 scores which indicated the severity of depressive symptoms. However, menopausal status was not found to be a significant risk factor for depression. These results can be explained by the fact that the KNHANES did not include questions about menopausal hormonal therapy, which may have weakened the association between depression and T2DM in our study.

According to several studies, higher PHQ-9 scores are associated with an increased risk of T2DM [37], diabetes complications [38], and poor self-management [39] in adult patients overall. However, this is the only study to have identified the relationship between diabetes progression status and depression in Korean midlife women using data from PHQ-9.

This study has some limitations. First, the KNHANES is a cross-sectional survey. Therefore, it is difficult to determine if T2DM directly causes depression in midlife women. Moreover, the KNHANES did not assess for various empirical factors (i.e., diabetes distress, self-efficacy, etc.) that are known to be covariates on both major variables; this may affect the lower explanatory power of regression model. Therefore, it is recommended to conduct further research to fully understand the interrelationships between T2DM, depression, menopause, and other health factors of the participants. Second, there is a potential selection bias as the parent study collected data from the community and excluded inpatients who admitted to hospitals. Additionally, we did not exclude individuals taking antidepressant medication.

Despite these limitations, the study has certain strengths. We used an objective standardized tool to measure depression in Korean women using nationally representative data. The study findings will therefore be useful for creating tailored approaches to diabetes care. These approaches should consider midlife women’s disease characteristics and the cultural and temporal aspects of their lives, including physical and psychological transitions.

## Conclusions

In the present study, we investigated the significant association between T2DM and depression in Korean midlife women using data from the KNHANES. Based on our findings, we recommend that routine screening for depressive symptoms be incorporated into the care of diabetes and pre-diabetes midlife Korean women, to ensure early identification and treatment of depression. Addressing their mental health will also help these women improve their diabetes self-management and glucose control. Furthermore, implementing these strategies could positively affect the overall health and quality of life on Korean midlife women with diabetes.

## Data Availability

KNHANES datasets are publicly accessible and freely available from: https://knhanes.kdca.go.kr/knhanes/main.do.

## List of abbreviations

T2DM: Type 2 diabetes mellitus
OECD: Organization for Economic Co-operation and Development
PHQ-9: Patient Health Questionnaire-9
KNHANES: Korea National Health and Nutrition Examination Survey
IRB: Institutional Review Board
WHO: World Health Organization
ADA: American Diabetes Association
FPG: Fasting Plasma Glucose

## References

[1] Heald AH, Stedman M, Davies M, Livingston M, Alshames R, Lunt M (2020). Estimating life years lost to diabetes: outcomes from analysis of National Diabetes Audit and Office of National Statistics data. Cardiovasc Endocrinol Metab.

[2] Jeong JH, Um YH, Ko SH, Park JH, Park JY, Han K (2017). Depression and mortality in people with type 2 diabetes mellitus, 2003 to 2013: a nationwide population-based cohort study. Diabetes Metab J.

[3] Egede LE, Ellis C (2010). Diabetes and depression: global perspectives. Diabetes Res Clin Pract.

[4] Bae JH, Han KD, Ko SH, Yang YS, Choi JH, Choi KM (2022). Diabetes fact sheet in Korea 2021. Diabetes Metab J.

[5] Sun H, Saeedi P, Karuranga S, Pinkepank M, Ogurtsova K, Duncan BB (2022). IDF Diabetes Atlas: global, regional and country-level diabetes prevalence estimates for 2021 and projections for 2045. Diabetes Res Clin Pract.

[6] Kim GE, Jo MW, Shin YW (2020). Increased prevalence of depression in South Korea from 2002 to 2013. Sci Rep.

[7] World Health Organization. Depression and other common mental disorders: global health estimates. 2017. https://apps.who.int/iris/handle/10665/254610. Accessed 25 Jan 2023.

[8] Katharine B, Robert CP, Richard IGH (2013). Antidepressant medication as a risk factor for type 2 diabetes and impaired glucose regulation: systematic review. Diabetes Care.

[9] Mezuk B, Eaton WW, Albrecht S, Golden SH (2008). Depression and type 2 diabetes over the lifespan: a meta-analysis. Diabetes Care.

[10] Chang SJ, Im E-O. Development of a situation-specific theory for explaining health-related quality of life among older south korean adults with type 2 diabetes. Res Theory Nurs Pract. 28(2):113–26. 10.1891/1541-6577.28.2.113. 10.1891/1541-6577.28.2.113 25087324

[11] Schram MT, Baan CA, Pouwer F (2009). Depression and quality of life in patients with diabetes: a systematic review from the european depression in diabetes (EDID) research consortium. Curr Diabetes Rev.

[12] Roy T, Lloyd CE, Pouwer F, Holt RI, Sartorius N (2012). Screening tools used for measuring depression among people with type 1 and type 2 diabetes: a systematic review. Diabet Med.

[13] Moriarty AS, Gilbody S, McMillan D, Manea L (2015). Screening and case finding for major depressive disorder using the Patient Health Questionnaire (PHQ-9): a meta-analysis. Gen Hosp Psychiatry.

[14] Centers for Disease Control and Prevention. Diabetes and mental health. 2022. https://www.cdc.gov/diabetes/managing/mental-health.html. Accessed 25 Jan 2023.

[15] Jang BN, Lee HJ, Joo JH, Park E-C, Jang S-I (2020). Association between health behaviours and depression: findings from a national cross-sectional study in South Korea. BMC Psychiatry.

[16] Im E-O, Yi J-S, Chee W (2021). Depressive symptoms and type II diabetes mellitus among midlife women. Menopause.

[17] Everson-Rose SA, Meyer PM, Powell LH, Pandey D, Torréns JI, Kravitz HM, et al. Depressive symptoms, insulin resistance, and risk of diabetes in women at midlife. Diabetes Care. 2004;27(12):2856–62. 10.2337/diacare.27.12.2856%J. 10.2337/diacare.27.12.2856 15562197

[18] Kravitz HM, Colvin AB, Avis NE, Joffe H, Chen Y, Bromberger JT (2022). Risk of high depressive symptoms after the final menstrual period: the study of Women’s Health across the Nation (SWAN). Menopause.

[19] Park H, Wenzel JA (2013). Experience of social role strain in korean women with type 2 diabetes. J Adv Nurs.

[20] Kim Y (2014). The Korea National Health and Nutrition Examination Survey (KNHANES): current status and challenges. Epidemiol Health.

[21] Inoue T, Tanaka T, Nakagawa S, Nakato Y, Kameyama R, Boku S (2012). Utility and limitations of PHQ-9 in a clinic specializing in psychiatric care. BMC Psychiatry.

[22] Erdfelder E, Faul F, Buchner A (1996). GPOWER: a general power analysis program. Behav Res Methods.

[23] World Health Organization. HEARTS-D: diagnosis and management of type 2 diabetes. https://www.who.int/publications/i/item/who-ucn-ncd-20.1. 2020. Accessed 20 Feb 2023.

[24] American Diabetes Association (2020). Addendum. 2. Classification and diagnosis of diabetes: standards of medical care in diabetes—2021. Erratum for: Diabetes Care.

[25] Park S-J, Choi H-R, Choi J-H, Kim KW, Hong JP (2010). Reliability and validity of the korean version of the Patient Health Questionnaire-9 (PHQ-9). Anxiety and Mood.

[26] Pan A, Lucas M, Sun Q, van Dam RM, Franco OH, Manson JE (2010). Bidirectional association between depression and type 2 diabetes mellitus in women. Arch Intern Med.

[27] Sung HN, Chae HS, Kim ES, Kim JS (2014). Diabetes and depressive symptoms in korean women: the fifth korean national health and nutrition examination survey (2010–2011). Korean J Fam Med.

[28] Park H, Kim MT (2012). Impact of social role strain, depression, social support and age on diabetes self-efficacy in korean women with type 2 diabetes. J Cardiovasc Nurs.

[29] Park SY, Cho S, Park Y, Bernstein KS, Shin JK (2013). Factors associated with mental health service utilization among korean american immigrants. Community Ment Health J.

[30] Zhou X, Min S, Sun J, Kim SJ, Ahn J-s, Peng Y (2015). Extending a structural model of somatization to South Koreans: cultural values, somatization tendency, and the presentation of depressive symptoms. J Affect Disord.

[31] Whitworth SR, Bruce DG, Starkstein SE, Davis WA, Davis TM, Bucks RS (2016). Lifetime depression and anxiety increase prevalent psychological symptoms and worsen glycemic control in type 2 diabetes: the Fremantle Diabetes Study Phase II. Diabetes Res Clin Pract.

[32] Robinson DJ, Coons M, Haensel H, Vallis M, Yale J-F (2018). Diabetes and mental health. Can J Diabetes.

[33] American Diabetes Association. Where diabetes meets depression 2022 [cited 2023 February 13]. Available from: https://diabetes.org/healthy-living/mental-health/are-you-experiencing-depression.

[34] Hur KY, Moon MK, Park JS, Kim S-K, Lee S-H, Yun J-S (2021). 2021 clinical practice guidelines for diabetes mellitus in Korea. Diabetes Metab J.

[35] Yu J, Lee SH, Kim MK (2022). Recent updates to clinical practice guidelines for diabetes mellitus. Endocrinol Metab (Seoul).

[36] Nouwen A, Winkley K, Twisk J, Lloyd CE, Peyrot M, Ismail K (2010). Type 2 diabetes mellitus as a risk factor for the onset of depression: a systematic review and meta-analysis. Diabetologia.

[37] Islam SM, Rawal LB, Niessen LW (2015). Prevalence of depression and its associated factors in patients with type 2 diabetes: a cross-sectional study in Dhaka, Bangladesh. Asian J Psychiatr.

[38] Ishizawa K, Babazono T, Horiba Y, Nakajima J, Takasaki K, Miura J (2016). The relationship between depressive symptoms and diabetic complications in elderly patients with diabetes: analysis using the diabetes study from the Center of Tokyo Women’s Medical University (DIACET). J Diabetes Complications.

[39] Nguyen AL, Green J, Enguidanos S (2015). The relationship between depressive symptoms, diabetes symptoms, and self-management among an urban, low-income latino population. J Diabetes Complications.

